# Complementary mechanisms for upright balance during walking

**DOI:** 10.1371/journal.pone.0172215

**Published:** 2017-02-24

**Authors:** Hendrik Reimann, Tyler D. Fettrow, Elizabeth D. Thompson, Peter Agada, Bradford J. McFadyen, John J. Jeka

**Affiliations:** 1 Department of Kinesiology, Temple University, Philadelphia, PA, United States of America; 2 Department of Physical Therapy, Temple University, Philadelphia, PA, United States of America; 3 Centre for Interdisciplinary Research in Rehabilitation and Social Integration, Université Laval, Québec, Canada; 4 Department of Rehabilitation, Université Laval, Québec, Canada; Tokai University, JAPAN

## Abstract

Lateral balance is a critical factor in keeping the human body upright during walking. Two important mechanisms for balance control are the stepping strategy, in which the foot placement is changed in the direction of a sensed fall to modulate how the gravitational force acts on the body, and the lateral ankle strategy, in which the body mass is actively accelerated by an ankle torque. Currently, there is minimal evidence about how these two strategies complement one another to achieve upright balance during locomotion. We use Galvanic vestibular stimulation (GVS) to induce the sensation of a fall at heel-off during gait initiation. We found that young healthy adults respond to the illusory fall using both the lateral ankle strategy and the stepping strategy. The stance foot center of pressure (CoP) is shifted in the direction of the perceived fall by ≈2.5 mm, starting ≈247 ms after stimulus onset. The foot placement of the following step is shifted by ≈15 mm in the same direction. The temporal delay between these two mechanisms suggests that they independently contribute to upright balance during locomotion, potentially in a serially coordinated manner. Modeling results indicate that without the lateral ankle strategy, a much larger step width is required to maintain upright balance, suggesting that the *small but early* CoP shift induced by the lateral ankle strategy is critical for upright stability during locomotion. The relative importance of each mechanism and how neurological disorders may affect their implementation remain an open question.

## Introduction

Upright balance is a critical factor of everyday human life, forming the basis for functional behavior like tool use. The neural control of balance has largely been studied under conditions of standing, with much of the analysis focusing on the different channels of sensory information detecting deviations in the kinematic state of the body and the modulation of the center of pressure (CoP) to correct them [1].

**Fig 1:**
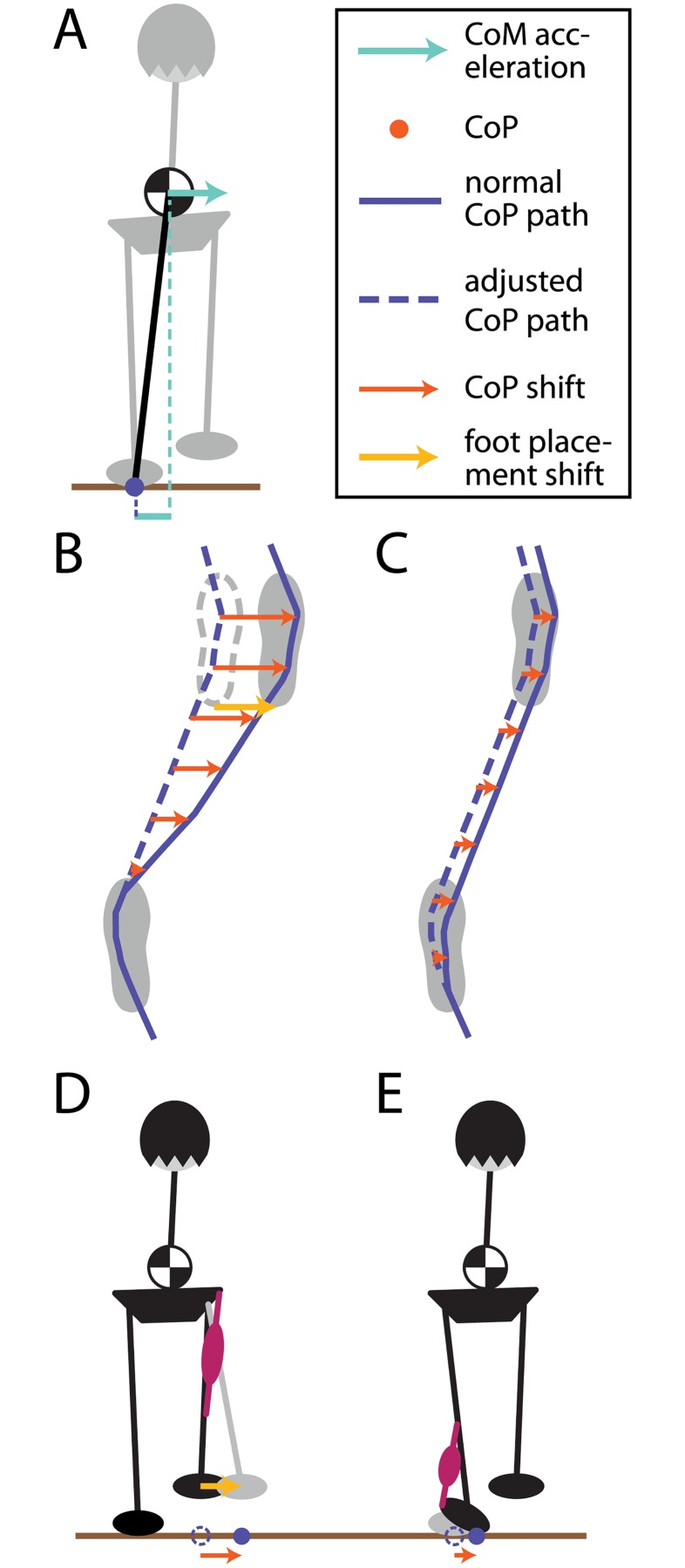
Mechanisms to maintain balance during locomotion in the frontal plain. Panel **(A)** shows the biomechanics of the upright body during locomotion under the simplifying inverted pendulum assumption. The horizontal CoM accelerates away from the CoP, with the magnitude of the acceleration proportional to the distance between the two. The lower panels illustrate how the CoP can be modified during walking. **(B)** At each new step, the lateral foot placement can be adjusted, shifting the CoP throughout the following stance phase. **(C)** During single stance, the CoP can be adjusted within the limits of the stance foot sole. **(D)** Foot placement changes are mainly accomplished by hip ab-/adduction of the swing leg, **(E)** lateral rolling of the stance foot by the ankle musculature.

Although the control problem in posture has often been simplified to a single degree of freedom in the inverted pendulum approximation, it has become clear in recent years that even in quiet stance, the entire kinematic chain contributes to balance [2, 3]. During locomotion, the inverted pendulum approximation is even less viable [4, 5], making the problem of understanding how balance is achieved during walking very challenging. Here we provide an avenue to understand the relative contributions of two mechanisms that are critical for upright balance during locomotion using Galvanic vestibular stimulation (GVS) as a perturbation to upright stance. The neural control mechanisms for upright balance during locomotion have received much less attention. Most research focused on the variation of the foot placement during steps. First, compared to standing balance, locomotion is more complex, both in terms of controlling the kinematic chain of the body, as well as behaviorally, since multiple subtasks are achieved in parallel (e.g. dynamic equilibrium, vertical support, foot trajectory, see [6, 7]). Moreover, while standing can be reasonably approximated with linear models, locomotion is inherently cyclic, leading to control requirements that change at different phases of the gait cycle (e.g. phase-dependent reflexes, see [8, 9]). Despite the differences between standing and locomotion, some of the biomechanical principles are the same. In the horizontal direction, the acceleration of the center of mass (CoM) is approximately proportional to its distance from the center of pressure (CoP) [10], as illustrated in Fig 1A. That means that any difference between the CoP and the CoM will lead to a fall if no control action is taken to correct it. The most extensively studied mechanism to affect the CoP location is to take a step (Fig 1B and 1D). Townsend first showed that modulation of the foot placement is already sufficient to control a simplified walker with point feet [11]. Subsequent research has focused on foot placement as a balance mechanism. Hof proposed a simple balance control principle, based on the extrapolated center of mass [12, 13], and showed that simulated trajectories with this controller are reasonably similar to experimental data. Donelan et al. measured kinematics during locomotion with passive lateral stabilization and observed reduced variability in lateral foot placement and metabolic cost, indicating that foot placement is indeed actively varied to maintain lateral balance [14]. Later work by Hof showed that humans respond to mechanical lateral perturbations by changing the foot placement in the direction of the push [15]. This response is preceded by activity in hip abductor muscles [16], suggesting an actively generated response. In a study of unperturbed gait, Wang and Srinivasan observed a correlation between the position and velocity of the pelvis in mid-stance and the following foot placement, indicating that such “stepping in the direction of the fall” is a regular mechanism of balance control in normal gait [17].

A second mechanism to affect the CoP is to apply an active muscle torque around the ankle of the stance foot (Fig 1C and 1E), similar to the ankle strategy in upright stance control [18]. This causes the foot to roll over and shifts the CoP across the foot sole The lateral ankle strategy has received relatively little attention in the human walking literature as a mechanism for controlling balance during walking. One reason for this is that compared to the stepping strategy, its ability to generate corrective balance responses seems far more limited. The available CoP excursions are ≈15 mm, constrained by the borders of the foot sole [15]. However, one distinct advantage is that the lateral ankle strategy can act much earlier than the stepping strategy, which has no effect before the next foot placement. There is some evidence that humans do indeed use the lateral ankle strategy during walking. Hof et al. reported that if the CoP deviates strongly from the average to one side at the beginning of a step, it tends to shift towards the opposite side during the step [19]. This “cross over” pattern of the CoP is missing in below-knee amputees, indicating that it is an active control mechanism depending on intact ankle musculature. Hof et al. also observed that in response to mechanical perturbations, the CoP shifts in the direction of the push, although it is difficult to discern how much of that shift is due to the push itself versus a neurally generated control action [15].

Another reason why change in step width is more extensively studied as a control mechanism of balance during locomotion is that it requires less active force generation than the ankle strategy [20], emphasizing the view that human mobility tends toward the most metabolically efficient solution. However, we argue that metabolic efficiency is a secondary concern in achieving stable and flexible upright posture during locomotion. Instead, completing the functional task at hand is crucial. For example, when performing a task such as carrying a bag of groceries inside one’s home, the nervous system is heavily invested in maintaining upright balance no matter the metabolic cost, not only from the standpoint of avoiding injury but also for the sake of completing the task as one navigates different support surfaces, doorways, etc. Indeed, it is important to note that maintaining upright balance is the basis for many of the phase-dependent reflexes observed during locomotion (e.g., [21]). From this perspective, rather than study these two balance mechanisms in isolation, our goal is to understand how they complement each other in achieving balance during locomotion.

In this study, we provide evidence that these two mechanisms are indeed used by the nervous system to maintain upright balance during locomotion. We perturb the locomotor control system by inducing the sensation of a fall using Galvanic vestibular stimulation (GVS) [22, 23]. Without the confounding effect of a mechanical perturbation, any observed changes must be induced by the neural controller instead of being an indirect mechanical effect of the perturbation. Importantly, we analyze the contributions of both CoP and step width shifts to understand how both mechanisms contribute to the overall balance response during locomotion using a computational model.

## Materials and methods

### Subjects

Nine healthy young adult subjects (4 male, 5 female, between 22 and 36 years of age, (30.2 ± 5.7), weighing 70.5 ± 14.8kg, volunteered for this study, including one of the authors (HR). Subjects were informed of the protocol and provided informed verbal and written consent to participate. Subjects had no self-reported history of neurological disorder or surgical procedures involving the legs, spine or head. The design was approved by the Temple University Institutional Review Board.

The sample size was determined according to a statistical power analysis. Based on pilot data, we expected an effect size of *d* = 0.24 for the step response. We chose the number of participants to reach an estimated power of 0.95.

### Experimental setup

The experiments were carried out by initiating gait from a Dual-top AccuSway force plate (AMTI Inc., Watertown, MA,USA) onto a walkway. Ground reaction forces were recorded from the force plates for each foot separately at 200Hz. Kinematic data were recorded at 120Hz using six Motion analysis Hawk cameras (Motion analysis Inc., Santa Rosa, CA, USA). Seven reflective markers with 20 mm diameter were attached to the Right and Left Antero-Superior Iliac Spines (R/L ASIS), the Midpoint between the Postero-Superior Iliac Spines (MPSIS), the fifth metatarsal heads (R/L MET5) and the tip of the second toes of the right and left feet (R/L TOE2). Binaural, bipolar GVS was delivered from two round electrodes with 3.2cm diameter (Axelgaard Manufacturing Co., Ltd, Fallbrook, CA, USA), placed on the mastoid processes behind the ears. GVS was triggered during the heel-off of the right foot, when the force measured by Force Sensing Resistor Sensor (Interlink Electronics Inc., Westlake Village, CA, USA) inserted below the heel in the right shoe dropped below a threshold. This threshold was individually determined for each subject as the average between two measurements, one during double stance and one with the right foot lifted. When triggered, a custom-made LabVIEW program (National instruments Inc., Austin, TX, USA) generated an analog control voltage from a National Instruments board (NI USB 6008 (National instruments Inc., Austin, TX, USA) which was transformed into a square wave of 0.3 mA current using a linear isolated stimulator (STIMISOLA, Biopac Inc., Goleta, CA, USA). The amplitude of the current was monitored using a current feedback monitor cable (CBLCFMA, Biopac, Inc.). The stimulus remained on while the subject was walking and was terminated by the experimenter after the fourth step. This relatively low level of current was chosen after pilot experiments to be high enough to induce a balance response, but not so high as to strongly affect the direction of movement.

### Test procedures

Participants stood on the force plate with their arms crossed and their eyes closed. The starting location of the feet was marked on the ground with adhesive tape during setup. After an oral command by the experimenter (“walk”), they initiated gait with their right foot and took four steps that led them off the platform onto the ramp, where they opened their eyes and the trial was concluded. GVS was delivered on heel-off of the right foot and continued for the full duration of the four steps. The polarity was changed so that the anode and cathode sides varied randomly between trials. To avoid confusion, we refer to the polarity conditions by their functional implications. This is the direction of the illusory fall, which coincides with the side of the cathode and is either to the LEFT or to the RIGHT. It is important to disambiguate this from the direction of the *actual* fall or lean usually observed in response to GVS, which is towards the anode, in the direction opposite of the illusory fall. This actual fall is a result of the actively generated motor response designed to catch the illusory fall. In a control condition, NO stimulation was delivered. Each of the three conditions was repeated 50 times, for a total of 150 trials. Conditions were randomized across all trials. A small number of the trials had to be excluded for some subjects because of problems with the visibility of the reflective markers. Breaks were taken after 30 trials, during which the subject sat down and rested, to avoid fatigue. Before data collection started, subjects went through the procedure several times until they felt comfortable with the GVS. Subjects were informed that GVS might perturb their balance and were asked to keep walking straight down the ramp. In case they felt severely unstable, subjects were instructed to open their eyes to prevent a fall, but no such case occured for any subject.

### Analysis

The kinematic data were filtered using a second order Butterworth filter with a cutoff frequency of 10Hz. Small gaps of up to 100ms in the marker trajectories due to occlusions were filled using cubic splines. Trials containing longer gaps were excluded from further analysis. To identify step events, we calculated the time derivative of the RTOE2 marker positions using finite differences. Pushoff/touchdown were defined as the first/last acceleration peak above a threshold of 3 *ms*^−2^.

For the CoP and RTOE2 marker trajectories during the step, time was normalized by resampling the data between stimulus onset and pushoff to 50 data points and the data between pushoff and touchdown to 100 data points. Similar to the foot placement, the response to GVS was isolated by subtracting the control mean for each subject at every data point. To estimate the onset times of the CoP and foot placement responses, we fitted a line to the response trajectories at the point of the maximal rate of change and defined the onset as the intersection of the two lines for the RIGHT and LEFT condition.

To analyze the foot placement response, we extracted the medial-lateral position, *R*_*ij*_ of the RTOE2 marker at the touchdown of the first step for each trial *i* and subject *j*. For each subject we calculated the step response *S* as the difference from the mean over the control trials (ctrl).

$$S_{ij}=\left(R_{ij}-{\bar{R}}_{\text{ctrl},j}\right)$$ (1)

We assume that in trials with GVS, this step response consists of three distinct components, all of which are reactions to perceived falls. The first component is the reaction to *actual* falls induced by natural fluctuations in the motor output previous to and during the step, i.e. motor noise, *S*^actual^. The second component is the reaction to the *illusory* falls induced by GVS, *S*^*GVS*^. The third component is the reaction to fall perceptions from natural fluctuations and inaccuracies of the sensory system, i.e. sensory noise, *S*^sensory^. We assume that the sensory component *S*^sensory^ is Gaussian white noise. The step response is then the sum

$$S_{ij}=S_{ij}^{\text{actual}}+S_{ij}^{\text{GVS}}+S_{ij}^{\text{sensory}}.$$ (2)

We further assume that the step response to the actual body movement depends linearly on the position *P*_*ij*_ and velocity ${\dot{P}}_{ij}$ of the pelvis at midstance, based on work by [17]. We use linear regression of the control trials to estimate this relationship with linear least squares as

$$J_{j}=S_{\text{ctrl},j}{\left(\begin{array}{c} P_{\text{ctrl},j}\\ {\dot{P}}_{\text{ctrl},j} \end{array}\right)}^{+},$$ (3)

where *J*_*j*_ is the 1 × 2 matrix of regression coefficients and *S*_ctrl,*j*_, *P*_ctrl,*j*_ and ${\dot{P}}_{\text{ctrl},j}$ are row vectors. This linear model allows us to estimate the actual fall response component in stimulus trials from the measured *P*_*ij*_ and ${\dot{P}}_{ij}$ and subtract it from the total step response to get an estimate of the GVS response as

$${\hat{S}}_{ij}^{\text{GVS}}=S_{ij}-J_{j}\left(\begin{array}{c} P_{ij}\\ {\dot{P}}_{ij} \end{array}\right).$$ (4)

### Statistical analysis

Statistical analysis was carried out in MATLAB 2014a, The MathWorks, Natick, 2014. For all tests, we used *α* = 0.05 as a threshold for statistical significance, correcting for multiple hypotheses where appropriate. We tested the foot placement in each condition for normality using a Shapiro-Wilkes test. We also tested for differences in foot placement variance between the stimulus and the control conditions using *F*-tests. To determine whether the foot placement response is different between conditions, we estimated the 95% confidence interval for the condition means, assuming the step response is normally distributed.

For the CoP-response, we tested for significant difference from zero with a one-tailed *t*-test for each condition at each time point. To account for multiple comparisons (300 total), we determined the *p*-values for each test using the method of [24] to control the false discovery rate (FDR) at a level of 0.05. This method is still valid when the comparisons are not independent, as is the case with time-series data [25].

### Modeling

We used the empirically measured responses in medial-lateral CoP and foot placement to fit a model of balance control during locomotion that includes both the foot placement and the lateral ankle torque mechanism. All parameter values are given in Table 1. The model was implemented in MATLAB 2014a, The MathWorks, Natick, 2014.

**Table 1:** Model parameters used in the simulations.

*α*	5	CoP modulation feedback gain
*γ*_*p*_	100 mm	GVS bias to head position sensor
*γ*_*v*_	10mms^−1^	GVS bias to head velocity sensor
*γ*_*a*_	10mms^−2^	GVS bias to head acceleration sensor
*σ*_*p*_	4.9 ⋅ 10^−2^ *mm*	standard deviation of head position sensor noise
*σ*_*v*_	3.7 ⋅ 10^−2^ *mms*^−1^	standard deviation of head velocity sensor noise
*σ*_*a*_	3.7 ⋅ 10^−2^ *mms*^−2^	standard deviation of head acceleration sensor noise
*d*	150 ms	neural time delay
Δ*t*	1 ms	Euler step
*l*	0.814 m	effective pendulum length
*σ*_step_	12 mm	standard deviation of foot placement noise
*σ*_init_	3.7 mm	standard deviation of initial foot position
*b*	5.5 mm	constant offset used by foot placement controller

#### Biomechanics

The body is modeled as a point-mass moving in a plane, supported by a single mass-less leg capable of instantaneous steps [12, 26]. The dynamics of this system depend only on the CoM position *p* and the CoP position *c*

$$\ddot{p}=\omega^{2}\left(p-c\right)$$ (5)

where $\omega =\sqrt{\frac{g}{l}}$, *l* is the height of the CoM above ground and *g* the acceleration from gravity. We define $x={\left(p,v,a\right)}^{T}$, where $v=\dot{p}$ and $a=\ddot{p}$ are the CoM velocity and acceleration, and $u=\dot{c}$, and translate this into the discretized system

$$x_{k}=Fx_{k-1}+Bu_{k},$$ (6)

with

$$F=\left(\begin{array}{ccc} 1 & \Delta t & 0\\ 0 & 1 & \Delta t\\ 0 & \Delta t\cdot \omega^{2} & 1 \end{array}\right),\;B=\left(\begin{array}{c} 0\\ 0\\ -\Delta t\cdot \omega^{2} \end{array}\right).$$ (7)

The control signal $u=\dot{c}$ represents the CoP modulation mechanism as defined below in Eq 12.

#### Sensor model

Observations of the system state are made each time step according to

$$z_{k}=x_{k-d}+\eta_{\text{sensor}},$$ (8)

where *d* represents a time delay and *η*_sensor_ is noise drawn from a multivariate normal distribution with zero mean and covariance matrix

$$C=\left(\begin{array}{ccc} \sigma_{p}^{2} & 0 & 0\\ 0 & \sigma_{v}^{2} & 0\\ 0 & 0 & \sigma_{a}^{2} \end{array}\right).$$ (9)

A Kalman filter combines these observations with a model of the system dynamics to form a time-delayed state estimate ${\tilde{x}}_{k}^{\left(d\right)}$ of the state at time *k* − *d*. The process noise for the Kalman filter is given by the motor noise term *η*_cop_ from Eq 12. Based on this time-delayed state estimate, a non-linear predictor then determines the best estimate ${\hat{x}}_{k}$ of the current state using an internal model of the body dynamics and the efferent copy of the control signal *u*_*k*_. For details about the Kalman filter equations and the non-linear predictor please refer to [27].

The illusory falls induced by GVS were modeled by adding biases *γ*_*p*_, *γ*_*v*_ and *γ*_*a*_ to the sensory estimates for *p*, *v* and *a*, so the CoM was perceived as leaning more to one side than it actually was.

#### Foot placement modulation

A step was taken after a fixed time interval *T*_step_ had elapsed. The position *R*_*n*_ of the *n*-th foot placement is determined according to *constant offset control* following [13].

$$R_{n}=\hat{\xi }+{\left(-1\right)}^{n}b+\eta_{\text{step}},$$ (10)

where

$$\xi =p+\frac{1}{\omega }v$$ (11)

is the *extrapolated center of mass (XCoM)*. The XCoM is a sum of CoM position and velocity, weighted by the natural frequency of the inverted pendulum (*ω*), first introduced by [12], and $\hat{\xi }$ is the estimate of *ξ* from the sensor model. The constant offset *b* is applied to alternating sides by the (−1)^*n*^, representing alternation between the left and right foot and *η*_step_ is Gaussian white noise representing uncertainties of lateral placement of the swing foot, i.e. one component of motor noise. The CoP position was re-iniatialized to *R*_*n*_ and an efference copy of *R*_*n*_ minus the unknown motor noise term *η*_step_ was used to re-intialize the sensor model at each step.

#### CoP modulation

During a step, the stance leg CoP is modulated according to a feedback law

$$\dot{c}=\alpha \left(\hat{\dot{\xi }}-{\dot{\xi }}_{\text{ref}}\right)+\eta_{\text{cop}},$$ (12)

where $\hat{\dot{\xi }}$ is the estimated rate of change of the XCoM and ${\dot{\xi }}_{\text{ref}}$ a reference value representing an expected value of $\dot{\xi }$ based upon efferent copies of prior motor commands and an internal model of the body dynamics. The *α* is a gain factor and *η*_cop_ is Gaussian white noise representing random fluctuations in the muscle forces affecting the CoP.

#### Expected XCoM reference

The efference copy of the foot placement command was used to generate a reference trajectory during each step. The rationale for this is that the CNS plans the foot placement to achieve a desired fall pattern for the next step, represented by a trajectory for the XCoM. We assumed that the desired trajectory of the XCoM is the one that would occur if the motor plan was executed flawlessly, i.e. without motor noise. For the *n*-th step, we initialized an internal forward model of the system dynamics to a state defined by the estimated body state at the time of the step, given by $\hat{p}\left(t_{n}\right),\hat{v}\left(t_{n}\right),\hat{a}\left(t_{n}\right)$, and the foot position given by *R*_*n*_. From these initial conditions, we integrated the internal model to calculate the reference trajectories *p*_ref_, *v*_ref_ and *a*_ref_, and from those ${\dot{\xi }}_{\text{ref}}=v_{\text{ref}}+\frac{1}{\omega }a_{\text{ref}}$.

## Results

Subjects were generally able to cope with the GVS perturbation to their balance, even in the absense of vision and with their arms crossed. Subjects experienced no adverse effects of the GVS during experiments such as nausea, headaches or falls. After removing trials with gaps due to occlusions, a total of 434 LEFT trials, 433 RIGHT trials and 464 trials with NO stimulation remained for analysis. The average time between stimulus onset and pushoff was 99 ± 31 ms (mean and standard deviation), average step time from pushoff to touchdown was 506 ± 58 ms.

### Experimental foot placement changes

The Shapiro-Wilkes tests failed to reject the null-hypotheses of normality for all three conditions (*p* = 0.057 for NO, *p* = 0.560 for LEFT, *p* = 0.144 for RIGHT). The *F*-tests failed to reject the null hypotheses of equal variances between the stimulus and control conditions in both comparisons (*p* = 0.605 for LEFT vs. NO, *p* = 0.962 for RIGHT vs. NO).

**Fig 2:**
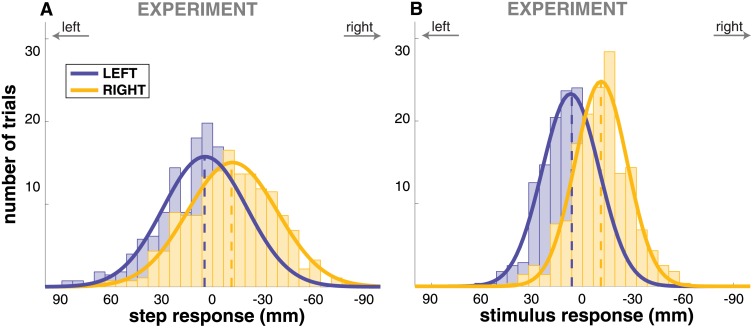
Histograms of the step response for the experimental data. Panel **(A)** shows the step response *S*, panel **(B)** shows the stimulus response ${\hat{S}}_{ij}^{\text{GVS}}$ for stimulus conditions LEFT (blue, *N* = 434) and RIGHT (yellow, *N* = 433). Solid lines are best fits with Gaussians, dashed lines indicate the mean for each stimulus condition.

Fig 2 shows histograms for the total foot placement response *S* and the estimated stimulus response, ${\hat{S}}_{ij}^{\text{GVS}}$. For illustration, we fitted the histograms with scaled Gaussians. The 95% confidence intervals for the step response are *S*_LEFT_ = [2.4, 7.4], *S*_RIGHT_ = [−13.6, −8.8], and for the stimulus response ${\hat{S}}_{\text{LEFT}}^{\text{GVS}}=[9.1,12.0]$, ${\hat{S}}_{\text{RIGHT}}^{\text{GVS}}=[-13.4,-10.3]$. In both conditions, the average foot placement is changed in the direction of the perceived fall. All confidence intervals exclude zero, indicating that lateral foot placement is an active response to the vestibular stimulus.

### Center of pressure modulation

**Fig 3:**
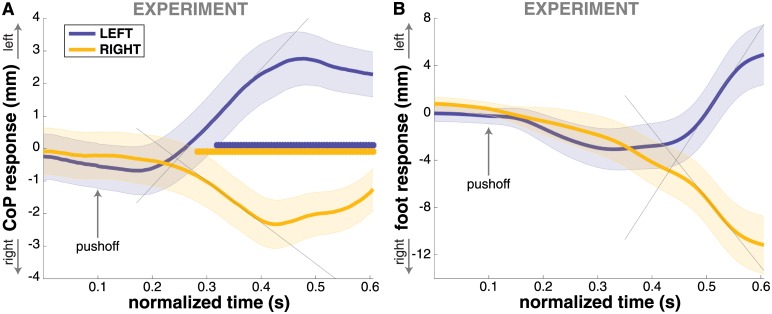
Response of the medial-lateral stance foot CoP and swing foot position from the experimental data. Panel **(A)** shows the CoP response, panel **(B)** the response of the swing foot heel. Normalized time ranges from stimulus onset to touchdown of the first step. Significant differences of the CoP response from zero at each time step are marked by blue (LEFT, *N* = 434) and yellow (RIGHT, *N* = 433) asterisks. The thin grey lines indicate the linear fits of the responses at the points of maximal rate of change, the intersection of these fits is the estimate of the response onset. Error bars indicate 95% confidence intervals.

The average CoP shift of the stance foot in response to GVS is shown Fig 3a. In both stimulus conditions, the CoP is shifted in the direction of the perceived fall. The magnitude of the CoP-modulation is slightly smaller when the illusory fall is to the RIGHT, but this difference is not statistically significant at any point in time (*t*-tests for each time step, FDR = 0.05).

The CoP shift onset is estimated at 247 ms after stimulus onset, as indicated by Fig 3a. In contrast, the laterial deviation of the swing foot starts 445 ms after stimulus onset, as shown in Fig 3b.

### Model simulation

**Fig 4:**
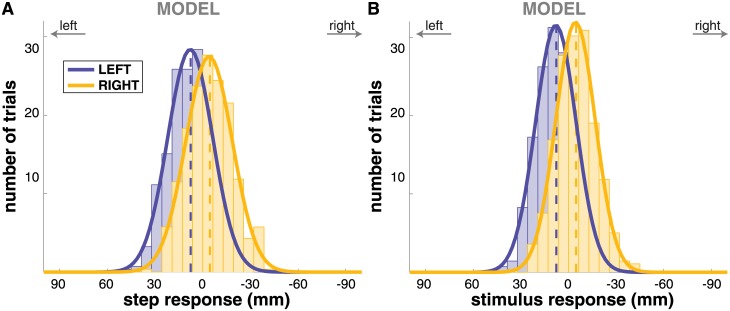
Histograms of the step response for the model simulations. Panel **(A)** shows the step response *S*, panel **(B)** shows the stimulus response ${\hat{S}}_{ij}^{\text{GVS}}$ for stimulus conditions LEFT (blue) and RIGHT (yellow). Solid lines are best fits with Gaussians, dashed lines indicate the mean for each stimulus condition.

**Fig 5:**
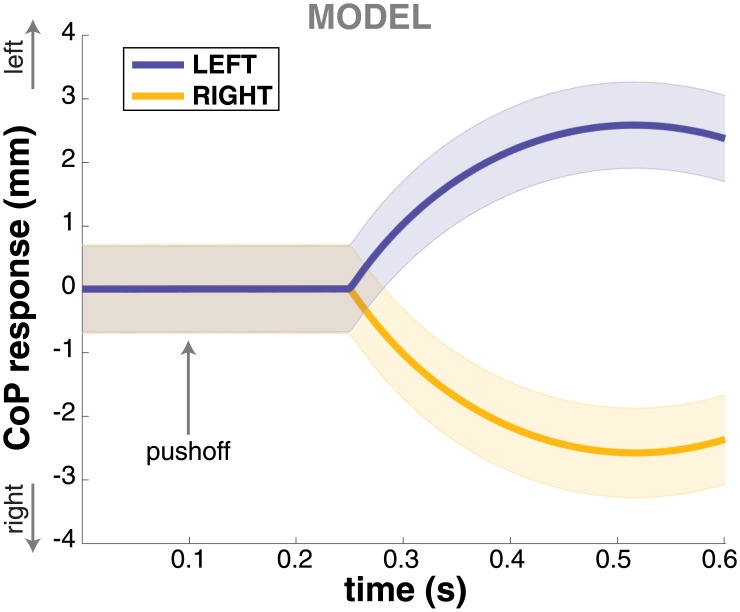
CoP response for the model simulations. Response of the medial-lateral stance foot CoP to LEFT (blue) and RIGHT (yellow) stimuli from the model simulations. Time ranges from stimulus onset to touchdown of the first step. Error bars indicate 95% confidence intervals.

We simulated *N* = 433 trajectories with the model in each of the three conditions and processed the simulated trajectories in the same fashion as the experimental data. Figs 4 and 5 show the step change and CoP shift response of the model. Both the step change and the CoP shift in the direction of the perceived fall are reproduced adequately by the model. The CoP shift generated by the model is not significantly different from the experimental data at any point in time (*t*-tests for each time-normalized data point, FDR = 0.05). The step change generated by the model is smaller than what is observed experimentally, however. This difference is statistically significant for both step response (*t*-test, *p* = 2 ⋅ 10^−6^) and stimulus response (*p* = 1 ⋅ 10^−12^) in the RIGHT condition, and for the stimulus response (*p* = 0.002), but not for the step response (*p* = 0.063) in the LEFT condition.

**Fig 6:**
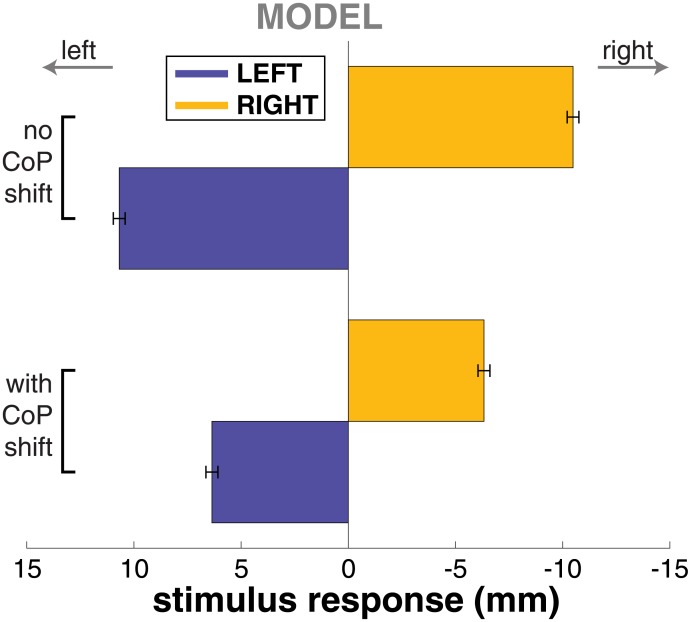
Step response changes with different mechanisms in the model. Step response to LEFT (blue) and RIGHT (yellow) stimuli from the model simulations. The top represents results from the controller without the CoP shift mechanism, the bottom results from the controller with the CoP shift mechanism.

To assess the effect of the CoP modulation mechanism, we also simulated the model with only the foot placement control. Fig 6 illustrates the resulting average foot placement response to the stimulus ${\hat{S}}_{ij}^{\text{GVS}}$ (bottom) in comparison to the data for the complete model with both mechanisms (top). The average step response of the model without the CoP modulation was about 65% larger than the step response of the model with CoP modulation.

## Discussion

We induced fall sensations to humans initiating locomotion using Galvanic vestibular stimulation (GVS), beginning at heel-off. We have shown that humans respond to the perceived falls with two distinct mechanisms. The first is a modulation of the stance foot center of pressure (CoP) in the direction of the fall. This modulation began about 247ms after the stimulus onset and lasted for the whole step. The second response is a shift of the foot placement of the following step in the direction of the perceived fall. These two mechanisms work in concert to achieve a more effective balance response than either one alone.

### Relative importance of the two mechanisms

The change in foot placement is effectively a shift in the CoP of the supporting limb throughout the next step. Both the CoP shift and the foot placement change in the direction of the fall induce CoM accelerations in the opposite direction, stopping the fall. At 10–15 mm, the magnitude of the step change is about 5 times as large as the initial stance foot CoP modulation, which peaks at 2.5 mm. One reason for this difference is that the ankle strategy is limited by the contact area between the foot and the floor. Accelerating the body by active modulation of the stance foot CoP requires an ankle torque. Changing the foot placement entails moving the swing leg in space, which requires only a comparably small hip torque [20]. The work of accelerating the body is then achieved by gravity instead of muscle torque.

However, the ankle strategy has the advantage of acting earlier than the foot placement strategy, because the latter only starts to accelerate the CoM after the next foot placement. This advantage in time may be crucial. Falls evolve quadratically in time, not linearly. A small correction applied early may effectively reduce the need for a large correction applied later. This notion is supported by the results of the model simulation, where adding the CoP control mechanism to the controller reduces the step response by approximately 40% (see Fig 6).

The role of each mechanism in response to a perceived fall might also depend upon the magnitude of that fall. It is possible that the relative contribution of each mechanism to prevent a fall shifts with the extent of the perceived threat to balance, e.g. that smaller perturbations are predominantly dealt with by the ankle strategy, while larger perturbations draw increasingly larger foot placement responses. Here, we only used relatively small stimulation levels of 0.3 mA, the dependence of each response on the stimulation level is subject to future study.

### Balance vs. navigation response to GVS

Several studies have examined the effect of Galvanic vestibular stimulation on locomotion before. Typical responses to GVS during walking are a combination of upper body lean and deviation of the walking path, both in the direction of the anode [23, 28]. [29] showed that blindfolded people in a wheelchair, with minimal balance demands, still show a deviation of the estimated traveling direction after GVS, suggesting that the navigation response is at least partially decoupled from the balance response.

The step response observed in our study appears to be a balance response, not a navigation response, because the overall path was experimentally constrained in our study. Although [29] showed with their wheelchair experiment that there is a navigation response that is decoupled from balance to some degree, We believe that the navigation response to GVS during normal gait is partially an indirect result of the foot placement strategy for balance control. To illustrate the rationale behind this, consider a GVS stimulus inducing an illusory fall to the right. The deviation in the first foot placement to the right, in response to this illusory fall to the right, leads to increased gravitational pull on the body to the left (see Fig 1). The result is an actual fall to the left during the following step, which in turn leads to a leftward shift of the second foot placement via the step response: The body is leaning to the left, so the foot has to step under it to prevent a fall. But because the GVS is still active and biases the estimate of the body state, this leftward shift is not sufficient to completely catch the leftward fall of the body, so this pattern repeats. The deviation of the walking path to the left emerges from this pattern of leftward body lean and leftward foot placement changes that catch the falling body.

### Relationship between responses in the upper and lower body

Bent et al. showed that the upper body lean toward the anode is independent of the phase of the gait cycle in which the stimulus starts during both gait initiation [30] and steady state walking [9]. The first instance of a change in foot placement, in contrast, appeared to be dependent upon the phase at which the GVS was delivered during the gait cycle in both of these studies. These authors concluded that there are two different responses to GVS, *(i)* the upper body balance response, which is phase-independent and appears with an onset of 300–400 ms for the head and 700–800 ms for the trunk, and *(ii)* the lower-body stepping response, which is phase-dependent and appears only at the *second* post-stimulus step. They argued that these findings suggest independent control of upper and lower body. A similar pattern of strong phase-dependency of the lower body and weak or no phase-dependency of the upper body was found in responses to visual scene movements by [31].

Our results, however, suggest that these observed responses in the upper and lower body might be closely related. Both the CoP shift and the foot placement change have relatively fast onset times (≈247 and ≈445 ms). These are in the same range as the head roll response of ≈300 ms reported by [9], and clearly faster than the trunk roll response (≈700 ms). Although caution is advised in directly comparing these numbers because of different methodology between our study and those of Bent et al. [9], we tentatively conclude that the CoP change, which is clearly a lower body response, occurs at least simultaneously with the upper body roll responses, if not earlier. More data is required to understand the relationship between the upper and lower body response in more detail.

### Origin of the CoP shift

The origin of the CoP modulation can emerge from numerous sources, but the current data set suggests ankle torque modulation. [29] reported short-latency responses (70–120 ms) in the leg muscles to GVS during upright stance. The relationship between stochastic vestibular stimulation and activation of the *gastrocnemius medialis* in both time and frequency domain was studied in detail by [32]. They identified a short-latency component (50–70 ms) and a medium-latency component (100–120 ms) of the response with opposite polarity. The CoP responses we observed started 247 ms after stimulus onset. The maximal excursion of the CoP occurred 478 ms after stimulus onset for LEFT and 427 ms for RIGHT, which is in general agreement with the numbers from Blouin et al. [32] considering the delay between muscle activation and force generation, supporting the notion that the CoP response is generated by muscle torques around the ankle, although comparisons should be made with caution considering the difference in methodology between these studies.

It is also possible that the CoP shift is a result of the upper body response to the GVS that generates the head and trunk roll reported by [9], who, as noted above, argued that control of the upper and lower body may be independent. It is important to point out, however, that even if the goal of this neural control strategy is to only move the upper body and leave the lower body unaffected, it would still need to activate the lower body musculature to cancel out the inertial interaction torques [33]. This inertial coupling between the body segments makes it hard to disambiguate upper body from lower body control from the available data.

It could also be argued that the CoP shift is a result of the lateral movement of the swing leg that leads to the change in foot placement. The ground reaction force generated by this movement has an effect on the CoP location. Also, the shift of the leg mass affects the whole body CoM, which factors into the CoP. However, the lateral movement of the swing foot leading to the step response starts ≈445 ms after GVS onset, which is about 200 ms later than the CoP shift (Fig 3). This difference in onset times indicates that these are two distinct phenomena and not merely two effects of a single mechanism. Although this evidence supports the notion of the step response and CoP shift being two independent mechanisms, it is likely that these are tightly coupled and temporally coordinated.

### Practical implications

It remains an open question how dysfunction in these balance mechanisms may lead to the common adaptations observed as individuals age or experience neurological trauma. For example, decreased gait velocity in older adults is common and one of the most powerful predictors of mortality for individuals over 70 years [34]. The slowing of gait is often attributed to weakness in calf muscles and is accompanied by increased step width and decreased stride length [35, 36]. Our results suggest that diminished ability to modulate the CoP during walking, potentially due to proprioceptive loss, may also contribute to increased step width in older adults. Without the ability to quickly sense the state (position and velocity) of the CoP under the stance foot, one must rely primarily on the step width mechanism by taking wider steps to maintain upright balance. Considering the importance of upright balance control during gait, unraveling the complementary nature of these balance mechanisms across different populations with poor balance control promises a new perspective on the priorities of the nervous system for functional mobility.

## Conclusion

We have provided evidence for the existence of two independent mechanisms for the control of upright balance during human locomotion, stance foot CoP manipulation and foot placement change. It remains to be seen how each mechanism changes its contribution to upright stability as we encounter different challenges such as an icy sidewalk or a sandy beach. Flexible coordination of these balance mechanisms may be the critical component for upright stability, which remains a challenge to define precisely during human locomotion.
